# The Dynamic Regulation of G-Quadruplex DNA Structures by Cytosine Methylation

**DOI:** 10.3390/ijms23052407

**Published:** 2022-02-22

**Authors:** Aaron John Stevens, Lucy de Jong, Martin Alexander Kennedy

**Affiliations:** 1 Department of Pathology and Molecular Medicine, University of Otago, Wellington 6021, New Zealand; dejlu879@student.otago.ac.nz; 2 Department of Pathology and Biomedical Science, University of Otago, Christchurch 8011, New Zealand; martin.kennedy@otago.ac.nz

**Keywords:** DNA methylation, G-quadruplexes, gene regulation

## Abstract

It is well known that certain non B-DNA structures, including G-quadruplexes, are key elements that can regulate gene expression. Here, we explore the theory that DNA modifications, such as methylation of cytosine, could act as a dynamic switch by promoting or alleviating the structural formation of G-quadruplex structures in DNA or RNA. The interaction between epigenetic DNA modifications, G4 formation, and the 3D architecture of the genome is a complex and developing area of research. Although there is growing evidence for such interactions, a great deal still remains to be discovered. In vivo, the potential effect that cytosine methylation may have on the formation of DNA structures has remained largely unresearched, despite this being a potential mechanism through which epigenetic factors could regulate gene activity. Such interactions could represent novel mechanisms for important biological functions, including altering nucleosome positioning or regulation of gene expression. Furthermore, promotion of strand-specific G-quadruplex formation in differentially methylated genes could have a dynamic role in directing X-inactivation or the control of imprinting, and would be a worthwhile focus for future research.

## 1. Introduction

DNA regulation is a complex process involving interactions among genomic, cellular, and environmental factors. As our understanding of genomic regulation develops, mapping how these factors interact to orchestrate tissue or cell-specific gene expression programmes remains one of the fundamental challenges for modern genetics. Epigenetic processes act at the interface between genetics and the environment, and include specific chemical modifications that can occur on the DNA, RNA, or histone proteins. In particular, the addition and removal of chemical groups to localised regions of DNA may have profound effects on the structural and functional state of DNA, and is likely to influence wide-scale genomic interactions. This process can influence gene regulation, and can direct cell specific gene expression by influencing chromatin structure and chromosomal organization to modify accessibility by the transcriptional machinery [1,2]. Although DNA largely exists in the well-recognised helical form (B-DNA), nucleotide bases can potentially interact in a variety of orientations. This enables the formation of non-canonical DNA (non B-DNA) structures, such as triplex, G-quadruplex, cruciform, hairpin, and *i*-motif structures [3,4,5]. Of these, G-quadruplexes (G4s) have been the most widely researched. In this review we focus our attention largely on G4 DNA structures and the evidence for impact of methylated cytosine in modifying formation of these structures, and to a lesser extent consider impacts of methylation on *i*-motif and H-DNA/triplex DNA structures. G4 structures have been recognised for many decades, and the study of their in vivo properties is now an important area of genetic research [6,7]. Emerging bodies of evidence are describing the role that G4s contribute to genomic processes relevant to gene maintenance, regulation, and DNA replication. DNA methylation is an additional layer of regulation that acts on the genome and the potential interaction between DNA methylation and G4 formation offers intriguing possibilities for dynamic regulation of these processes.

In mammalian genomes, 5-methyl cytosine (5mC) is arguably the best understood of the epigenetic modifications. It is involved in directing cell specific gene expression [8,9,10], expression from imprinted genes, X-chromosome inactivation, transposon silencing [11,12], and is an important regulator of DNA conformation [13]. The pattern of 5mC in genomic DNA from different cell lineages (the ‘methylome’) is largely established during early gestation [14] and methylation differences in different cell lineages partly control cell differentiation [15]. However, the dynamic addition and removal of methylation from cytosine also provides a mechanism through which gene activity can be modulated in response to environmental cues. Subtle changes to the human methylome continue to occur throughout adulthood, and many environmental factors including smoking [16], pollutants [17,18], stress [19,20], exercise [21,22], and alcohol consumption [23] have been strongly associated with changes in the adult methylome. Additionally, aging [24] and disease development such as diabetes and heart disease [25,26] are strongly associated with certain methylation profiles, and drastic alterations are a hallmark of most cancers [27,28]. 

The formation of 5mC in DNA involves DNA methyltransferase (DNMT) enzymes, which recognize CpG dinucleotides and catalyse the addition of a methyl group to the fifth carbon of a cytosine, converting it to 5mC [29]. The methyl group is positioned in the major groove of DNA where it does not interfere with Watson–Crick base pairing, but can modulate the binding of various proteins to DNA [30,31]. The genomic occurrence of CpGs in mammals is less frequent than expected, and the majority of CpGs are sparsely distributed through genic and intergenic genomic regions. These tend to be highly methylated, whereas CpGs found in dense GC-rich clusters called CpG islands (CGIs) are mostly depleted of methylation [12,32]. Despite being a small chemical modification, 5mC can have a variety of effects on DNA stability, chromatin structure, and accessibility of DNA to transcription factors. By promoting the formation of tightly packed chromatin, this in turn regulates gene activity by influencing whether regions of DNA are accessible to factors and proteins required for the initiation of transcription [33,34,35]. However, it is important to recognise that the function of DNA methylation is context dependent, and the correlation between DNA methylation and transcription is more complex than initially anticipated [32]. 

Although research is demonstrating correlative links between DNA methylation changes and disease, mechanistic investigation into how subtle DNA methylation changes can direct phenotypic outcomes has been limited. Likewise, our understanding of how cytosine methylation can inhibit or promote DNA–protein binding, or influence DNA structure, is also poorly understood. In many instances the genomic position at which altered DNA methylation is observed does not correspond with regulatory elements, and may often occur within intergenic regions, making the biological significance difficult to interpret. It is often concluded that such changes could influence the expression of genes through distal gene interactions, and changing the three dimensional (3D) organization of the chromosome. Although Hardin et al. was the first to observe that cytosine methylation may aid in the formation of secondary DNA structure [36], the implications of this are rarely discussed in a genomic context. The interaction between epigenetic DNA modifications, non B-DNA formation, and the 3D architecture of the genome is a complex and dynamic area, and although there is developing evidence for such interactions, a great deal still remains to be discovered. In vivo, the potential effect that cytosine methylation may have on promoting or hindering non B-DNA formations has remained largely un-researched, despite this being a potential mechanism through which epigenetic factors could regulate gene activity. Such interactions could represent novel mechanisms for important biological functions, such as the regulation of gene expression, altering nucleosome positioning (stable G4s induce subsequent genomic rearrangements), or the control of genomic imprinting.

### 1.1. G4 Formation and Cytosine Methylation

**Figure 1:**
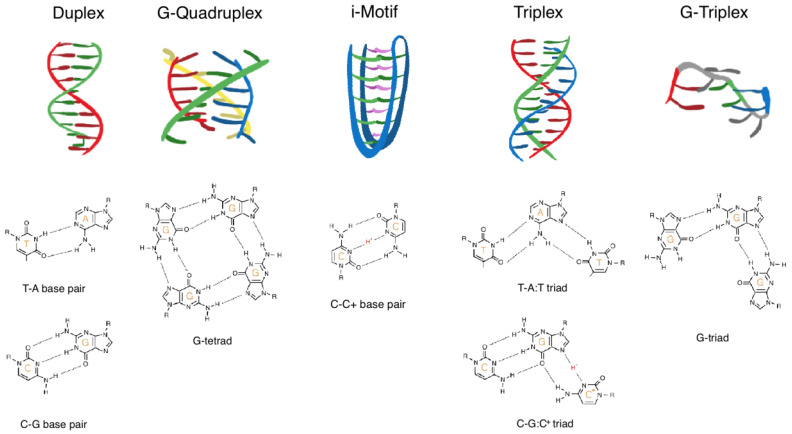
Schematic illustration of structural DNA motifs. From left: duplex, G-quadruplex, *i*-motif, triplex, and G-triplex. Examples of the contributing base pairing pattern is depicted below each motif: T-A and C-G for the duplex, G-tetrad for the G-quadruplex, C:C^+^ for the *i*-motif, T-A:T and C-G:C^+^ for the triplex, and G-triad for the G-triplex.

Guanine is unique among the four nucleoside bases due to its ability to self-associate through Hoogsteen–hydrogen bonds between four nucleotides. This results in a stable, square planar arrangement referred to as a G-tetrad, which forms the bases of a G4 structure (Figure 1). Two or more stacked G-tetrads are then connected by a linker of nucleotides that are not normally involved with the tetrads themselves [37]. The general basic pattern of a G4-forming DNA sequence involves two or more guanine repeats that are separated by up to seven linking nucleotides, repeated four times within a motif e.g., (GGGN_(1-7)_(3)-GGG). Although the traditional description of a G4 consists of between one and seven linking nucleotides, the maximum number of linkers is an estimate [38]. The propensity of dsDNA sequence to adopt a G4 structure and the subsequent structural stability is context dependent and determined by multiple factors, however, the main determinants are the strength of the competing Watson-Crick base pairing, and the ionic conditions. The presence of a positively charged monovalent cation (typically potassium) stabilizes the G4 by neutralising the negative charge of inward facing O-6 oxygen atoms. The incorporation of different cations into a G4 structure often promotes the formation of distinct G4 topologies, and divalent cations such as magnesium have also been shown to have diverse and unpredictable effects on both topology and stability [39,40,41,42]. The in vivo formation of G4 [43,44] and their many potential topological variants have been extensively reviewed [37,45,46], as have their biological roles [47,48,49]. 

Largely due to their high propensity for formation, and their potential application as drug targets, especially for cancer, G4 structures have received more research activity than other non-canonical DNA structures. This also extends to the in vitro characterisation of how DNA methylation influences G4 structural properties. Consequently, there are reports of varied effects of DNA methylation on structural conformation, stability, and the molecular association times of different G4-forming sequences. Bioinformatic prediction algorithms can be used to perform genome-wide, in silico analyses of the location of putative G4 motifs, and have indicated that CpGs are generally precluded from co-localisation with quadruplex motifs. This suggests that instances of overlap may be detrimental and therefore selected against [50], or that their combination might serve a specific biological function. Although the in vivo effects of 5mC on G4 formation are in the preliminary stages of research, anecdotal evidence suggests that many of the observed in vitro properties may reflect similar in vivo properties.

### 1.2. Cytosine Methylation Stabilises Non Watson-Crick Base Pairs

DNA methylation is known to alter the molecular dynamics in double stranded B-DNA by decreasing backbone flexibility, and increasing thermal stability [51,52,53], an effect that may be attributed to direct interactions of the methyl group with adjacent bases and adjacent methyl groups [54]. Using circular dichroism spectroscopy, the chemical stabilisation of G4 by methylation was first demonstrated in 1993 by Hardin et al. [36]. This effect was attributed to non-Watson-Crick pairing between two cytosine bases in a G4-forming sequence, where the addition of 5mC greatly increased the stability and kinetic associations of the G4 structure, even though they did not directly contribute towards the guanine-based Hoogsteen bonding [36]. The cytosine-to-cytosine bond involves protonation of one cytosine base (C·C^+^), which facilitates hydrogen bonding, allowing for formation of three hydrogen bonds and enabling cross-linking between two DNA strands (Figure 1) [55,56]. This can occur between opposing cytosine bases in G4 structures, and it was concluded that cytosine methylation may alleviate the structural requirement for protonation in cytosine:cytosine bonds, aiding in the formation of secondary DNA structures [36]. This observation was later reinforced by Lin et al. [57] and a similar mechanism of stabilisation was also documented for *i*-motif and DNA triplexes [57,58] (Figure 1).

Cytosine base pairing forms the basis for the formation of *i*-motif DNA structures, which are composed of intercalated and hemi-protonated C·C^+^ base pairs in a head-to-tail orientation [59]. The hemi-protonated nature of the C·C^+^ bond has a base-pairing energy, which is actually stronger than the canonical Watson-Crick G·C base pairing, however, the neutral C:C base-pairing energy is substantially less [59]. Because *i*-motifs require cytosine repeats in nucleic acids they have a high propensity to form in DNA sequences that are complementary to G4s [60,61,62]. However, unlike G4 topology, *i*-motif formation is highly reliant on the protonation of cytosine. Despite early predictions that 5mC would destabilize non B-DNA structures, empirical evidence suggests that there is generally a stabilizing effect, especially for *i*-motif structures [63,64]. The addition of only a single methyl site has been repeatedly observed to increase the thermodynamic stability of *i*-motifs by approximately 10 °C, with further modifications having less pronounced effects [65], and hypermethylation resulting in destabilisation. This appears to be consistent regardless of whether the methyl group is positioned in the core or loop [66], however, the stabilisation effect does appear to be most pronounced at near-physiological pH [66]. Therefore, 5mC in dsDNA may act on both DNA strands to promote non B-DNA formation by favouring G-quadruplex structures on one strand and *i*-motif structures on the complementary C-rich strand. 

DNA triplexes are three stranded structures involving two DNA strands that are bound through Watson-Crick hydrogen bonds, with a third single-stranded, purine-rich DNA strand bound to the dsDNA through Hoogsteen bonds (Figure 1). Similarly to the *i*-motif, the triplex formation is also stabilised by pH and 5mC, and, similar to G4, cation presence also plays a role in determining stability [67]. Replacing cytosine with 5mC within a triplex-forming strand has the effect of allowing the third strand to bind at a physiologically relevant pH [68] and raises the thermal stability of the structure [58]. Few papers have further explored these observations beyond these initial observations, or have attempted to verify the original mechanistic proposals [69]. However, the evidence is supportive of similar observations with *i*-motif and G4 structures where the C·C^+^ base-pairing is stabilised by DNA methylation. This can influence the progression of polymerases along DNA during replication and transcription through regions of methylated DNA [70]. It may also represent a novel method of gene silencing at differentially methylated genes (or the X-chromosome in females), where RNA polymerase is trapped in the bubble by the folding of the third RNA strand [70]. This is a similar concept to the formation of R-loops, which can arise during transcription of the 5′-UTR in G-rich DNA. In this instance, the newly synthesized RNA invades the upstream dsDNA and forms a three stranded RNA:DNA hybrid with the template strand, by displacing the non-template strand. This has been demonstrated to free up the non-template strand, increasing the propensity for G4 formation and enhancing transcription [71]. Similar to G4 structures, R-loops are also enriched in promoters, unmethylated CpG islands, and may also prevent methylation of the underlying DNA sequence (discussed below) [72], which has been proposed as a potential form of epigenetic regulation and transcription termination [73,74].

### 1.3. Cytosine Methylation and G4 Formation in RNA

RNA modifications are widely prevalent and chemically complex; however, there is currently a limited knowledge of the function of RNA modifications and their effect on RNA structure and function [75,76]. The in vivo formation of the G-quadruplex in RNA has been demonstrated [77,78], where it likely functions as a regulator of translation and impacts on protein binding [79]. This has been demonstrated in plants, where the RNA G4 directs development and growth and is likely to be involved in novel functions, such as post-transcriptional regulation of gene expression [78]. This is a developing area of genetic research and represents one of the next big challenges for understanding gene regulation [80]. It is plausible that many of the potential effects of 5mC on DNA G4 structures discussed in this review will also have a similar underlying function in RNA G4 structures and may play a dynamic role in regulating translation.

### 1.4. In Vitro Effects of G4 and DNA Methylation

One of the most widely recognised features of G4 is the ability to arrest polymerase during DNA amplification [81,82,83], which has been attributed to several different types of PCR failures, and allelic drop-out (ADO) [84,85]. During PCR amplification of a differentially methylated gene locus, we found that the combination of cytosine methylation and G4 formation can have profound effects on amplification efficiency, which leads to allelic drop-out of methylated DNA during PCR [86]. Similar observations were subsequently made by Yoshida et al., who used qPCR to demonstrate reduced amplification efficiency of methylated G4 motifs when compared to non-methylated G4 motifs [87]. At these gene regions, methylation appears to cause subtle changes in thermodynamics and the kinetic properties of DNA, such that the presence of 5mC on the maternally methylated DNA copy promotes the rapid reformation of G4 structures during PCR, inhibiting *Taq* polymerase for a period sufficient to cause a complete amplification bias of the paternal DNA [88,89,90]. This observation of ADO is likely to be of wider relevance to imprinted genes that contain at least one G4 motif, and, without stringent genotyping controls, could easily go undetected [90]. It is also likely that this form of ADO may influence next-generation sequencing preparations [91] and, theoretically, RNA sequencing, as both G4 and 5mC methylation are commonly found in RNA. Our observation of decreased DNA G4 stability with 5mC [86] is contrary to similar studies [57], which likely reflects differences in the experimental conditions used. However, our observations that 5mC impacts transitions between duplex or hairpin DNA and non B-DNA formation have been replicated by others [65,92]. It is also likely that the effect of 5mC on G4 stability is dependent on additional parameters such as ionic environment, G4 topology, G4 sequence, the position and number of methylated cytosine within the G4 region, and molecular crowding [93]. 

### 1.5. Potential In Vivo Effects of G4 and Methylation

In the instances where DNA methylation modulates the stability or propensity of non-B-DNA formation [36], this may hold potential for harnessing as a targetable property in cancer therapeutics. G4 sequences have been demonstrated to occur more frequently near transcription start sites, telomeres, ribosomal DNA, immunoglobulin heavy-chain switch regions, and CpG islands [48,94,95,96]. G4 formation in promoter regions has generally been associated with transcriptional suppression, and this has been demonstrated with several proto-oncogenes [97,98,99,100], including *RET* [101], c-*MYC*, *BCL*-2 [102], and *VEGF* [103]. However, there is also evidence that gene expression can be enhanced by the selection of alternative G4 conformations [104]. The potential interaction between 5mC and G4 structure could represent an additional layer of regulatory control, where addition or removal of methylation from G4 structures could regulate gene expression or enzyme recruitment by altering G4 structural potential or topology. Although it is widely accepted that 5mC can change the structural properties of DNA by reducing DNA flexibility, which may aid in promoting chromatin formation [105], the ability of 5mC to change the chemical and physical properties of DNA has received surprisingly limited research.

Transcription factors perform the first step in decoding the genome by directing gene expression and chromatin structure through protein–DNA interactions at specific genome locations [106,107]. Based on in vitro binding studies, it has been demonstrated that binding of transcription factors can be affected by cytosine methylation [107,108] and numerous transcription factors are recruited to sites of G4 formation [109]. Several transcription factors that bind to G4 structures can have high binding affinities, which are comparable to that of canonical DNA double-strand interactions. Furthermore, G4s in gene promoters (especially for highly expressed genes) also appear to be bound by a large number of transcription factors, such as SP2, E2F4, NRF1, or FUS [110,111]. The ability of DNA methylation to direct both transcription factor binding and formation of G4 structures could represent a mechanism for the dynamic regulation of transcription, especially in directing cell specific transcription programmes. Recent research is supporting this role, and has suggested that G4s may act as an epigenetic mark responsible, at least in part, for the recruitment of SP1 [52].

One of the few in-depth analyses into the mechanistic effects of DNA methylation on G4 formation, demonstrated that 5mC in CTCF transcription factor binding sites promotes quadruplex formation. In the instance of the *hTERT* gene, this prevented CTCF from binding and lead to increased expression [92]. It was demonstrated that 5mC alone was not sufficient to inhibit CTCF binding to the first exon of *hTERT*, which suggested that G4 formation (promoted by CpG methylation) inhibited CTCF binding and further regulated gene expression [92]. It is reasonable to suggest that these effects occur in vivo and could impact on the process of epigenetic gene regulation. In the instance of *hTERT*, these findings provided mechanistic insight into how hypermethylation of an oncogenic promoter can lead to expression in most telomerase-positive tumors. 

DNA replication is a highly regulated procedure, where replication originates at multiple sites across genomic DNA, with certain sites associated with early and late timing. These origins of replication have been demonstrated to be intricately linked to G4 formation, where the number of G4 on each strand appears to influence the efficiency and timing of individual origins [112]. G4-based origins were subsequently demonstrated to be mainly localised in non-coding regions with low epigenetic marks, yet a high level of DNA methylation within the G4 forming motif [113]. Similar to the widely recognised ability of G4 to hinder DNA amplification by *Taq* polymerase, DNA replication through G4-forming regions is also not straightforward and often requires recruitment of specific helicases such as Pif1, Wrn1, FANCJ, BLM, RTEL1, and DDX11 that resolve G4 structures [114,115,116,117,118,119].

Faithful replication through G4 structures requires a highly conserved multistep mechanism of G4 resolution, which has only recently been resolved [120]. Stalling of DNA polymerase during replication can induce single stranded breaks, making G4 forming regions hot-sports for genomic rearrangements [121,122,123]. Arrest at G4 sites on the leading template strand during replication has been demonstrated to partially delink replication from repackaging of newly made chromatin. This can result in errors in copying parental histone modifications, and can compromise epigenetic memory [124]. Formation of G4 structures is generally suppressed in the heterochromatin of human cells, with their presence associated with dynamic epigenetic features in chromatin and correlated with genes showing elevated transcription [125]. Given that 5mC also promotes the formation of chromatin [126], this effect could be additionally enhanced by the combination of methylation and G4 structures, or on the inactivated X chromosome. Conversely, G4 have also been demonstrated to promote replication by recruiting helicases, and it has been proposed that Reversionless1 (REV1), a key enzyme involved in aiding replication through sites of DNA lesions or damage (translesion synthesis), acts at G4 motifs formed at the replication fork [124]. Given the further complications for *Taq* polymerase caused by the interaction of 5mC and G4 formation, it would be worth investigating whether these properties are enhanced in vivo in areas containing both G4 and DNA methylation.

It has been proposed that G4 structures could provide signals which direct the enzymatic activity of methyltransferases [127], which are known to have a higher affinity for unusual structures in DNA relative to B-form DNA [128]. During DNA replication, DNA methyltransferase enzymes (DNMT) transfer the pattern of DNA methylation from the parental strand to the newly synthesized daughter strand. This process ensures the faithful transfer of tissue specific DNA methylation patterns across cellular generations. It has been demonstrated that G4s are a genomic feature that direct methylation at CpG islands [109], which may explain the observation that CpG islands predominantly lack 5mC [1,129]. G4 sites are enriched for DNMT1 binding, which support previous hypotheses that high affinity of DNMT1 for binding G4 has a sequestering effect, thereby preventing certain CpG islands from becoming methylated [109]. In this instance, CpG islands in active chromatin that contained a G4 structure were depleted in methylation and the surrounding flanking regions displayed higher than average methylation. This suggests that G4s may play an important function in the establishment of the epigenome [109]. DNA methyltransferases can also carry out *de novo* methylation to create new methylation patterns, and there is evidence that DNA structures can act in vivo to initiate or block *de novo* methylation in adjacent DNA. It has been proposed that double stranded DNA may not be the primary substrate for *de novo* methylation. Instead, single stranded structures formed during DNA replication may serve as nucleation sites for *de novo* methylation of adjacent DNA regions [130]. It has also been hypothesised that G4 structures could be involved in maintaining epigenetic signatures through several cycles of replication [46,124]. These observations draw several parallels with R-loop formation [72,73,74], and given the high propensity for overlap between R-loop and G4 forming DNA sequences, these two factors may act in unison, or through similar mechanistic paths.

Imprinted genes are a subset of genes, which are monoallelically expressed and display differential gene methylation depending on the parental origin of the alleles. Imprinted gene clusters are unusually rich in CpG islands [131] and these differentially methylated regions (DMRs) frequently overlap with CpG islands. Thus, CpG islands of imprinted genes may contain special DNA elements that distinguish them from CpG islands of biallelically expressed genes. Likewise, the inactivated X-chromosome and imprinting control regions (ICRs), also contain methylation on one chromosomal copy, determined by the parent of origin. The methylated copy in such regions tends to a correspondence with the inactivation of that gene, although the processes that lead to this are not fully understood [131,132]. Minor changes in the methylation levels of ICRs often cause substantial errors in the imprinting of the corresponding domain, reinforcing the integral role methylation plays in ICRs [133]. It has previously been suggested that G4 formation may play a role in enzyme recruitment at DMRs [124]. However, based on the differential effect that methylation can have on G4 propensity, it seems logical that this could also drive selective enzyme recruitment towards a single parental (either methylated or non-methylated) DNA strand. Although it has received limited scientific investigation, it is possible that G4 formation plays a fundamental role in directing gene silencing at imprinted genes, which are often associated with differential methylation. Our observations that methylation in DMRs can substantially alter G4 propensity, and hinder *Taq* polymerase, may be relevant to processes of DNA replication or transcription.

### 1.6. Potential for Novel G4 Formation in Cancer Due to Abnormal Methylation

Abnormal DNA methylation changes are a ubiquitous observation in cancer and an important factor in tumour development and progression [134,135]. For example, key genes involved in promoting cell growth and division often have less methylation in tumour cells when compared with ordinary tissue, which results in their over expression. Alternatively, genes involved in directing cell apoptosis pathways often have increased methylation leading to gene silencing in cancer cells [136]. It has been observed that a characteristic of solid tumours is the occurrence of large hypomethylated blocks of genomic DNA [137]. Hypomethylation in regions of high G4 propensity has been suggested to cause genomic hotspots for recombination, by inducing double-stranded DNA breaks [138,139,140], which could be a factor that drives cancer development [50]. This has been hypothesized to bridge the roles of genetic and epigenetic influences directing tumorigenesis [138]. Furthermore, a disproportionately high incidence of G-quadruplex motifs has been observed in the promoters of oncogenes, in contrast to the promoters of tumour suppressors which exhibit an extremely low G-quadruplex formation potential [97]. These abnormal methylation and expression profiles, which are a frequent characteristic of cancer, could provide an environment for formation of G4 structures not otherwise expected elsewhere in the human genome, outside of the tumour environment. This may lead to the selective formation or inhibition of key regulatory G4 structures that drive oncogenic gene expression, and provide a substrate for therapeutic targets. G4 structures have been extensively investigated as novel drug targets in cancer therapeutics [141]. Characterising the in vivo effects of DNA methylation on G4 structures may provide a possible therapeutic avenue to further enhance specificity in the tumour environment. Additional DNA modifications, such as 8-oxoguanine [142] or methylation at CpA dinucleotides [143] can affect the structural kinetics of non B-DNA formations and have been linked to the silencing of cancer genes in lymphoma and myeloma cell lines [144]. However, the effect that these could have on non B-DNA structural formation has not attracted substantial scientific investigation. Further investigations into the role that DNA methylation could play in directing RNA-mediated G4 structures or the formation of regulatory R-loops could also play a role in selective lethality in cancer cells, and further progress research towards considering G4s as therapeutic targets in human diseases [79].

## 2. Conclusions

There is a growing body of literature supporting the idea that methylation of G4 structures may be of fundamental importance to genome structure and function, playing an integral role in directing regulatory G4 formation in gene promoters and also directing wider establishment of epigenetic marks. Despite having substantial biotechnological, therapeutic, and biological relevance, the potential for 5mC to regulate formation of G4 structures has received relatively little scientific attention. This hypothesised additional layer of regulation could allow for methylated G4s to act as a dynamic epigenetic switch, selectively activating or repressing gene expression in a cell specific or environmental context. Furthermore, the potential influence of methylation on G4 formation in differentially methylated gene regions may facilitate parent of origin gene expression at imprinted genes or in the instance of X-inactivation. There is also substantial scope for selective therapeutic applications in the context of the tumour environment.

## Data Availability

Not applicable.
